# Hydrogen sulfide mitigates spatial learning deficits in angiotensin II-Induced hypertension via inhibition of endothelial NOX2 and neuroinflammation

**DOI:** 10.1038/s41398-026-04178-0

**Published:** 2026-06-20

**Authors:** Gaurav Kumar, Shalini Mishra, Shyamal Goswami, Manish Sharma

**Affiliations:** 1 School of Life Sciences and Biotechnology, Chhatrapati Shahu Ji Maharaj University, Kanpur, UP 208024 India; 2 Defence Institute of Physiology and Allied Sciences (DIPAS), Defence Research and Development Organization (DRDO), Delhi, India; 3 Department of Internal Medicine, Wake Forest University School of Medicine, Winston Salem, North Carolina USA; 4 School of Life Sciences, Jawaharlal Nehru University (JNU), New Delhi, India

**Keywords:** Molecular neuroscience, Physiology

## Abstract

Hypertension (HTN), when prolonged, extends beyond the cardiovascular system, impairs neurovascular function, and progresses into cognitive impairment. Thus, it is essential to understand the mechanism that drives HTN-induced brain pathogenesis for devising novel therapy. This study explored spatial learning, vessels-associated microglia and neuroinflammation during angiotensin II (Ang II) induced HTN with the hypothesis that after HTN induction, endogenous H_2_S level contributes to neuropathology in HTN. Male Sprague Dawley rats received Ang II (600 ng/kg/min) or saline using an implanted osmotic pump, and NaHS (4 mg/kg), an H_2_S donor was given intraperitoneally 10 days prior and during Ang II infusion. We employed H_2_S estimation by amperometric probe, spatial learning was assessed through the Morris water maze task, and synaptic proteins were probed in a synaptosomal fraction of hippocampus homogenate using Western blot. Additionally, rat cytokine array and immunofluorescence were used to screen for chemokine and cytokine, and the endothelium microglial phenotype, respectively. The pretreatment of NaHS attenuated Ang II-induced spatial learning and perturbation of synaptic protein in the hippocampus. It is associated with significant reduction in endothelial activation and microglia pro-inflammatory phenotype in both parenchymal and vessel-associated microglia. Moreover, above observation is accompanied by decreased proinflammatory cytokines and chemokines, such as, TNF-α, MCP-1, VEGF, and IL-1ɑ, among others. In summary, our data demonstrated that maintenance of H_2_S level attenuated HTN induced spatial learning deficit by suppressing neuroinflammation and endothelial NOX2 expression. Therefore, reinforcing the role of H_2_S in alleviating oxidant-induced inflammatory injury and eventually maintaining brain function.

## Introduction

Hypertension (HTN) continues to be a major public health concern worldwide [1]. The pathophysiological effects of hypertension are not confined to cardiovascular system, also perturbs neuropathological conditions in the brain, such as stroke and vascular dementia [2, 3]. Notably, systolic blood pressure intervention trial – memory and cognition in decreased hypertension (SPRINT MIND), showed that keeping blood pressure under strict control decreases the risk of mild memory problems and also reduce the risk of dementia [4]. However, knowledge of the underlying mechanisms and therapy of hypertension-induced cerebrovascular damage remains limited. Hypertension impairs neurovascular control accompanied with abnormal intracranial pressure (ICP) and blood-brain barrier (BBB) dysfunction [5, 6].The physiological mechanisms contributing to hypertension primarily include oxidative stress, which evolves into neuroinflammation [7, 8].Oxidative stress may serve as both a trigger and a consequence of immune system imbalance and neuroinflammation. This cascade of event contributes to vascular injury, remodeling, and the progression of hypertension [9, 10]. Recent evidence has shown NADPH oxidase 2 (NOX2) as a central mediator of reactive oxygen species (ROS) production, as well as its activation promoting neuroinflammation [11, 12].Furthermore, accumulating evidence confirms that an activated inflammatory response and the immune system play a critical role in the development and progression of hypertension. These processes are also associated with hypertensive complications such as cerebral small vessel disease, hemorrhagic stroke, and dementia [13].

Previous studies have described low-grade inflammation characterized by microglial activation, and elevated production of pro-inflammatory cytokines (IL-1β, IL-6, and TNF-α) in hypertensive brain regions [14, 15].The perpetuation of neuroinflammation in HTN involves paracrine signaling within the neurovascular unit (NVU), which includes cerebral blood vessels and other cell types such as microglia, astrocytes, neuron among others [16]. Interestingly, dynamic interactions between endothelial cells and microglia in response to vascular injury operates in a biphasic manner during brain injury, in early phase, microglia exert a protective role [17, 18]. However, chronic microglia stimulation is highly activated, deleterious phenotype that secretes pro-inflammatory cytokines, chemokines, and proteases [19]. A clearer understanding of the relationship between Ang II-induced hypertension and neuroinflammation is pertinent. Also, understanding of cognitive impairment in the context of the NVU is essential for developing novel therapeutic approaches for hypertension-induced brain pathology.

As HTN induced pathology is multifactorial in nature potential interventions may include drugs that reduce vascular damage, microglial activation, oxidative stress, and proinflammatory molecule levels. Hydrogen sulfide (H_2_S), an endogenous gasotransmitter, exerts pleiotropic effects under various physiological conditions [20]. Moreover, recent studies have shown that H_2_S supplementation provides significant benefits to the circulatory system, attenuates brain vascular aging, and improves cognitive function [21, 22]. While animal studies have confirmed a critical pathological role for endogenous H_2_S deficiency in the development of hypertension, the role of the H_2_S axis in regulating neurovascular responses and its underlying molecular and cellular mechanisms in hypertension remains poorly understood [23].

In this context, the present study aimed to investigate the role of H_2_S in modulating hypertension-induced cognitive deficits. We used a systemic Ang II infusion model to induce hypertension which is a model of secondary hypertension. Moreover, sodium hydrogen sulfide (NaHS) was administered as an H_2_S donor. The study specifically aims to evaluate learning function, alterations in brain H_2_S homeostasis, neuroinflammatory responses, microglial activation, vessels associated microglia and changes in synaptic protein expression in Ang II infusion model of HTN.

## Methods

### Animals and treatment groups

All experimental procedures were approved by the Institutional Animal Care and Use Committee and adhered to the guidelines of the Committee for the Purpose of Control and Supervision of Experiments on Animals (CPCSEA), Government of India. Male Sprague-Dawley rats (230–250 g) were used in this study and were kept under standard laboratory conditions at the Institute’s animal facility. Rats were randomly divided into four groups: Control, Ang II (14 days), Ang II (14 days) + NaHS, and NaHS alone using simple randomization. Sample sizes were selected based on previous studies using similar Ang II–induced hypertension models and pilot experiments to detect biologically meaningful differences. To minimize animal use 3 R principles (Replacement, Reduction, and Refinement) were followed. Animals were housed in filter-top cages (4 per cage; floor area: 821 cm²; Cat. No. SRC02, Orchid Scientific & Innovative India Pvt. Ltd., India) under controlled environmental conditions (22 °C, 54–60% humidity, 12 h light/dark cycle), with ad libitum access to standard pellet diet and water. All animals were habituated to the experimenter before behavioral testing and sacrifice. Animals were included following screening for anxiety like behaviors and general health status. Animals were excluded only in cases of unexpected mortality, severe illness, or technical failure during sample processing. No outliers were removed from the dataset during statistical analysis. Behavioral experiments were conducted between 9:00 a.m. and 11:00 a.m. To induce hypertension, Sprague- Dawley rat received Ang II (230–250 g, 600 ng/kg/min) infusions via a subcutaneously implanted Alzet osmotic minipump (Model 2004) for 14 days [24]. Rats in the NaHS-treated groups received intraperitoneal (i.p) injections of sodium hydrogen sulfide (NaHS·xH_2_O, Cayman Chemicals, dissolved fresh in 1.5 M PBS (pH 7.4)) at 4 mg/kg body weight daily for 10 consecutive days prior and during Ang II infusion. Sham controls received equivalent volumes of PBS. Systolic blood pressure (SBP) was assessed in all the groups at baseline and on 3, 7 and 14 days by tail-cuff plethysmography (NIBP System, ADInstruments). To reduce the effects of stress caused by this method on blood pressure measurement, the animals were habituated for one week before the actual measurement began.

### Amperometric probe-based estimation of H_2_S

Rats were anesthetized with ketamine (50 mg/kg) and xylazine (10 mg/kg, i.p.) and transcardially perfused with ice-cold 0.01 M PBS to eliminate blood, and brain samples were collected. Dissected brain tissue was snap-frozen in liquid nitrogen and homogenized at 40 mg/mL in deoxygenated 0.01 M PBS containing 50 μM diethylenetriamine pentaacetic acid (DTPA). The collected supernatant was used for H2S estimation using the polarized sensor. H_2_S levels in brain tissues were quantified using an amperometric H_2_S-specific sensor (ISO-H2S-100-CXX) connected to a free radical analyzer (TBR1025, World Precision Instruments, USA) [25, 26]. The probe was polarized in 0.01 M PBS to achieve a stable baseline current (0.1–2 nA). Calibration was performed using sodium sulfide (Na_2_S) at increasing concentrations (100, 200, 400, 800, and 1600 nM). Each sample was tested in at least three dilutions, and H_2_S levels were recorded from a minimum of five animals per group and presented as bar graphs.

### Zinc precipitation assay for sulfide estimation

The zinc precipitation assay was also used to quantify levels of sulfide as earlier reported [27]. Cerebral tissues were homogenized to 40 mg/mL in 50 mM sodium carbonate buffer (pH 9). In 500 µL homogenate 350 µL of 1 percent zinc acetate and 50 µL 1. 5 M NaOH were added. On centrifugation (5000 rpm, 4 °C, 12 min), precipitate zinc sulfide was filtered off and washed with water and the centrifugation was repeated. A pellet was dissolved in 160 μL of water and 40 μL of a 1:1 blend of dyes 20 mM NN-dimethyl-p-phenylenediamine in 7.2 M HCl and 30 mM FeCl 3 in 1.2 M HCl) was added. The reaction mixture was then left to incubate at room temperature in a 10- min interval, and absorbance was read at 670 nm. λmax was confirmed by full spectral scans (500–700 nm). Reagents were all made up in degassed Milli-Q water.

### Morris water maze (MWM) test

Spatial learning memory was measured by MWM as outlined above [28]. Over 5 successive days, rats were trained to find a submerged platform in circular water tank. The rats were expected to get four trials each day with an interval of 5 min. In each of the trials, each of the rats was released in a distinct quardent and they were given the opportunity to locate the platform at the bottom of quadrant Q1. Thereafter, upon reaching the platform, rats had a 30 s stay, after which they were dried and put back to the home cage. Rats that did not locate the platform in one minute were partly escorted to the platform. An overhead camera linked up to the ANY-maze video tracking system (Stoelting, USA) was used to monitor behavioral performance. Various Parameters such as the latency (time taken to arrive at the platform) and path length (distance covered), and swimming speed were measured and compared between the group.

### Synaptosome extraction

Synaptosomes were isolated using Syn-PER Reagent (87793, ThermoFisher) as per the manufacturer’s instructions. Briefly, 100 mg of brain tissue was homogenized in 1 mL Syn-PER Reagent on ice using a Dounce homogenizer (~10 strokes). The homogenate was centrifuged at 1400 g for 10 min at 4 °C to remove debris, and the supernatant was further centrifuged at 15,000 g for 20 min at 4° C. The resulting pellet contained synaptosomes used for protein extraction.

### Western blotting

30 μg of protein extracted from brain tissues were separated by SDS-PAGE and transferred to nitrocellulose membranes. After blocking with casein blocking buffer (B6429, Sigma-Aldrich) overnight at 4 °C, membranes were subsequently incubated with the primary antibodies at specified dilutions, namely N-methyl-D-aspartate receptor 2B subunit gene (NMDAR2B, ab28373, Abcam) at 1:1500 working dilution, PSD95 (ab192757, Abcam) at 1:1500 working dilution, Synaptophysin (ab8049, Abcam) at 1:1500 working dilution; AT1R (ab124734, Abcam) at 1:1500 working dilution; NOX2/gp91phox (ab80897, Abcam) at 1:1500 working dilution; NOX2/p67phox (ab109366, Abcam), β-Actin (SAB2100037, Sigma) at 1:5000 for 2 h at 25 °C. This is followed by incubation with suitable HRP-conjugated secondary antibody (A0545, Sigma) at 1:15000 or 1:20000 working dilutions. Membranes were incubated with HRP-conjugated second antibodies after being washed. Detection of protein bands was performed with an enhanced chemiluminescence (ECL) detection kit. SuperSignal™ West Femto Maximum Sensitivity Substrate, Thermo Scientific, 34095). Using ImageJ software (https://imagej.nih.gov/ij/), normalized mean intensities (specific target/internal control) were calculated to determine the relative expression among different groups and represented as bar graphs (summarizing data from 3 independent experiments).

### Immunofluorescence

Rats were anesthetized (ketamine,50 mg/kg body weight and xylazine, 10 mg/kg body weight, i.p) and perfused with cold PBS and then followed by 4% paraformaldehyde in PBS. Under-processed brains were post-fixed in 4% paraformaldehyde. For immunofluorescence analysis, brain tissue was equilibrated in ascending concentration of sucrose(10, 20% for 24 h and 30% for 48–72 h).To ensure optimal immunostaining, brain sections were washed with PBST (PBS in Triton X-100 0.1%), heat antigen retrieval was conducted with Sodium citrate buffer (pH 6.0 and 0.05% Tween-20). The sections were blocked with 5% goat serum after which they were incubated with the desired primary– Iba1 (ab178846, Abcam) at 1:200 working dilution; vWF (ab6994, Abcam) at 1:500 working dilution; CD31 (ab182981, Abcam at 1:500 working dilution; CD68 (ab201340, Abcam) at 1:200 working dilution; antibodies overnight at 4 °C) ; NOX2/gp91phox (ab80897, Abcam)at 1:200 working dilution Alexa Fluor 488-conjugated anti-mouse and Alexa Fluor 594-conjugated anti-rabbit secondary antibodies (Invitrogen) were also used. The nuclei were counterstained with DAPI (diluted 1:1000; Sigma-Aldrich) diluted into 2 mg/mL stock solution. The gelatin-based mounting media were used to mount sections. At least 3 section per animal (*n* = 3) were examined, with 2–5 randomly selected fields per section. Images were acquired using an Olympus BX61 fluorescence microscope with a digital camera and analyzed with CellSens Dimension1, and ImageJ software (NIH) (https://imagej.nih.gov/ij/). ImageJ plugin JACoP was employed for co-localization spatial association based on Pearson’s correlation with consistent thresholding across groups. Thresholds were set using background subtraction and identical acquisition settings.

### Cytokine array

Cytokine expression was assessed using the Abcam Rat Cytokine Antibody Array Membrane containing 34 targets (ab133992, Abcam). Experimental procedure for detecting cytokine and chemokine were performed according to the manufacturer’s instructions. Cytokines detection membrane was blocked by 1X blocking buffer and incubated overnight with the same quantity of total protein (200 μg) from different groups (three biological replicates per group). Blocked membranes were washed 3 time with provided wash buffer and incubated with biotinylated antibody (2 hr, room temperature). Subsequently, membrane was incubated with horseradish peroxidase (HRP)- conjugated streptavidin (1:1000) (additional 2 hr at room temperature). Signal detection was performed via chemiluminescence, following the manufacturer’s protocol. Developed membranes were scanned and spots were analyzed using ImageJ software (NIH). To determine relative expression of each cytokine and chemokine were normalised with the internal control provided within the array.

### Statistical analysis

Statistical analysis was performed using Graph Pad Prism 8.0.2 (Graph Pad Software, CA, USA). Data are presented as mean ± SEM. Behavioral and image Analyses were performed in a blinded manner. Statistical significance was determined using one-way analysis of variance (ANOVA), followed by Bonferroni post hoc test for multiple comparisons. Variance between groups was assessed during analysis and was comparable across the groups being statistically compared. All data were presented as means ± S.E.M. *p < 0.05, **p < 0.01 and **p < 0.001 were considered significant; n.s. indicates not significant.

## Results

### Pretreatment with NaHS in brain maintains H_2_S levels in angiotensin II -Induced hypertension

**Fig. 1:**
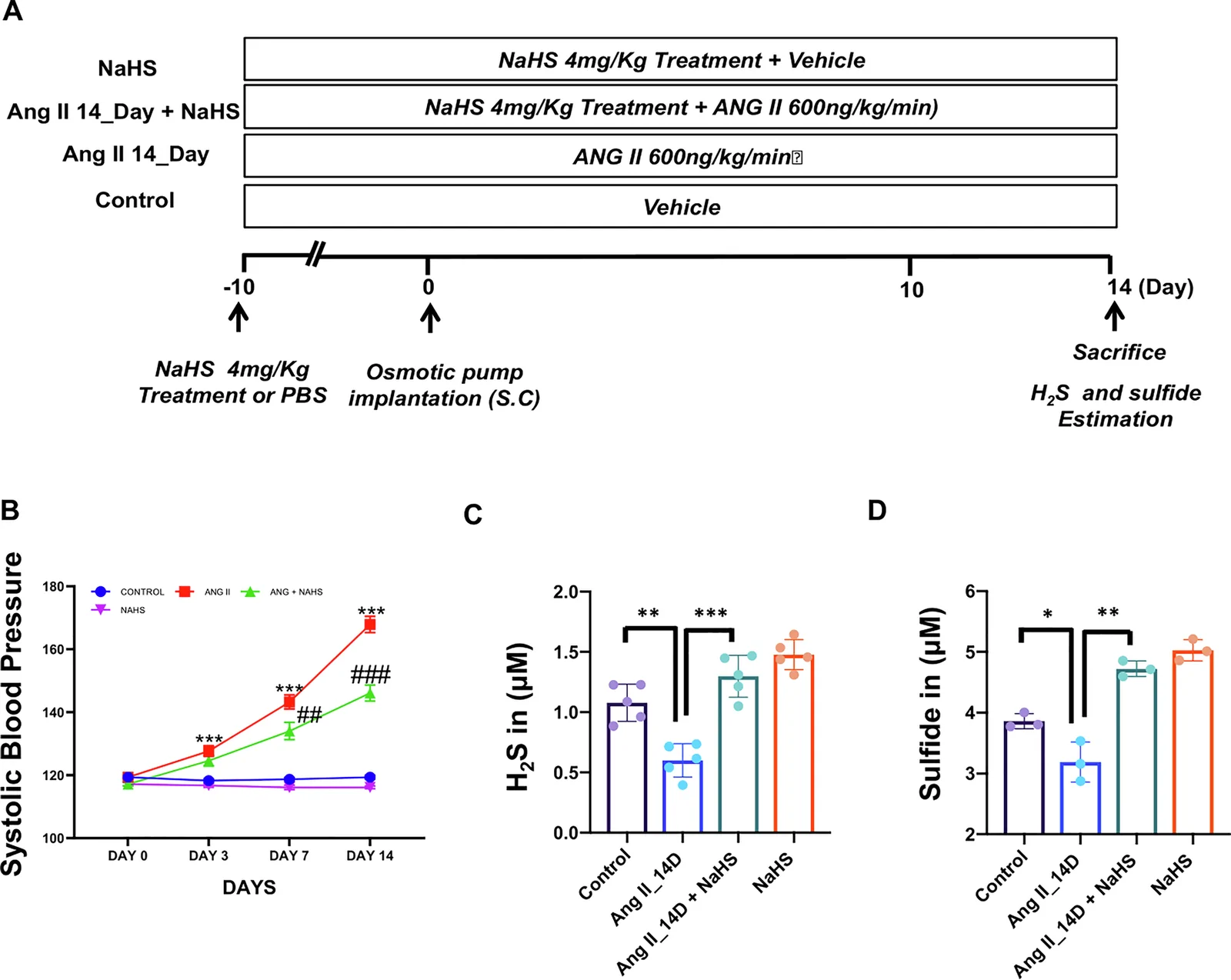
Pretreatment with NaHS in brain maintains H_2_S levels in Angiotensin II-Induced Hypertension. **A** Schematic diagram of the experimental design showing treatment groups in the current study. Animals from control, angiotensin II (600 ng/kg/min) plus vehicle, angiotensin II plus NaHS (4 mg/kg); saline plus NaHS (4 mg/kg) groups were subjected to the Morris water maze test. **B** line graph showing systolic blood pressure measured at day **0, 3, 7, and 14** in the **control, Ang II, Ang II** + **NaHS NaHS**, and groups. Day 0 value (2 day before osmotic pump implantation).Data are presented as **mean** ± **SEM**. **C** Scatter with Bar graph representing levels of H_2_S (in μM) in brain tissue extracts of animals in mentioned groups (saline), (n = 5). **D** Scatter with Bar graph representing concentration of total sulfide (in μM) in brain tissue extracts of animals groups as mentioned (*n* = 3) * indicates statistical significance of **Ang II** compared with control group, while **#** indicates statistical significance between the **Ang II** and **Ang II** + **NaHS** groups. *p < 0.05, **p < 0.01, ***p < 0.001, ^#^*p* < 0.05, ^##^*p* < 0.01, ^###^*p* < 0.001.

Hypertension was induced in this study by subcutaneous administration of Ang II (600 ng/kg/min) using an ALZET osmotic pump (model 2004) for 14 days. Moreover, NaHS (an H₂S donor) at a dose of 4 mg/kg/day was administered for 10 days prior to osmotic pump implantation and continued during the Ang II infusion. A schematic diagram of the experimental protocol is shown in Fig. 1A. Ang II infusion (600 ng/kg/min) significantly induced a sustained increase in systolic blood pressure (SBP) at 3, 7, and 14 days. Pretreatment with NaHS significantly attenuated the Ang II–induced rise in blood pressure; however, SBP remained higher than in normal control animals. No significant changes in SBP were observed in the control or control + NaHS groups throughout the study period (Fig. 1B).

H_2_S being an important gasotransmitter modulating the circulatory system, still how endogenous H_2_S levels are perturbed in the brain following systemic hypertension has remained incompletely understood [29]. We therefore examined H_2_S and sulfides concentration in the brain in all the above mentioned groups. H_2_S was measured employing an amperometric H_2_S-specific probe from brain tissue under different conditions as per the published [30]. The H_2_S sensor (ISO-H_2_S-100-CXX) was connected to a free radical analyzer (TBR1025, World Precision Instruments, USA) and utilized to measure the H_2_S level. The test measures H_2_S concentration at the sensor tip, directly proportional to the electrochemical current it produces. As shown in (Fig. 1C) we observed a significant decrease in the levels of H_2_S in response to Ang II infusion post-14-day infusion. The pretreatment of animals with NaHS resulted in a marked increase in H_2_S levels in brain under both Ang II infusion and vehicle groups. We next utilised the Zinc Precipitation Assay, a modified methylene blue assay that involves an additional zinc precipitation step to trap H_2_S. This test quantifies total Sulfides levels in brain tissue, which directly correlates with the H_2_S level. As shown in (Fig. 1D) sulfide was significantly increased in the brains of rats pre-treated with NaHS in Ang II infused model and vehicle group as compared to the only Ang II infusion group.

Therefore, ANG II infusion provided an opportunity to investigate the relationship between maintenance of H_2_S level in brain and cognitive function specifically learning along with associated neuroinflammation, cerebral vasculature, and microglial responses under these conditions.

### H_2_S protects spatial learning impairment and maintains synaptic protein expression in angiotensin II -Induced hypertension

We evaluated the effect of NaHS pretreatment on hippocampus-dependent spatial learning deficits in Ang II-induced hypertension, we employed the MWM test. This behavioral paradigm assesses spatial learning by measuring the latency and path length required to locate a hidden platform over multiple training days.

**Fig. 2:**
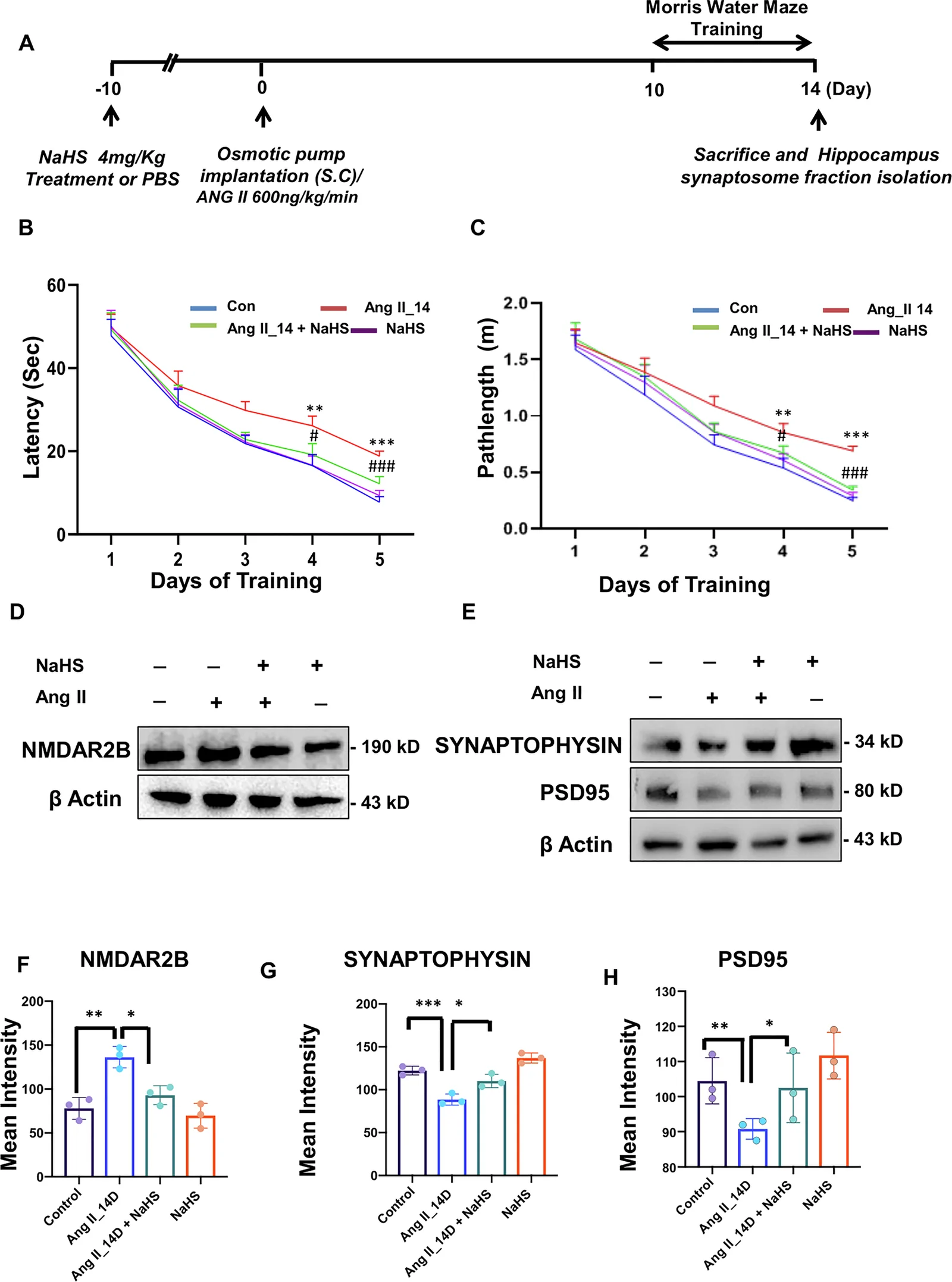
H_2_S protects spatial learning impairment and maintains Synaptic Protein Expression in Angiotensin II -Induced Hypertension. **A** Schematic diagram of the experimental design of the Morris water maze test. The animals were habituated to water for 2 days and, subsequently, trained to learn in the Morris water maze for 5 days. Line graphs representing day-wise changes in (**B**) latency (Second) and (**C**) path length (Meter) over the 5-day training period of animals in the Morris water maze. **D** Representative western blots of NMDAR2B, (**E**) PSD95 and synaptophysin in the Hippocampal samples obtained from the indicated groups. Scatter with Bar graph showing normalized protein expression of (**F**) NMDA R2B (**G**) Synaptophysin & (**H**) PSD95 Mean ± SEM is shown. *p < 0.05, **p < 0.01, ***p < 0.001, ^#^*p* < 0.05, ^##^*p* < 0.01, ^###^*p* < 0.001.

The MWM Test is described in detail in the methods section and timeline of experiment is shown in (Fig. 2A). Animals were trained to find a hidden platform in a pool of water in this activity. Prior to the training phase, the animals were exposed to the MWM to guarantee that they had the best visual acuity and motor capacity to escape, regardless of their ability to learn. All the animals in the training trial swam and discovered the hidden platform normally. As shown in Fig. 2B, C, the time it took the animals to reach the platform (latency) and the length of the path they travelled (pathlength) to get there decreased with each passing day during the 5-day training regimen, in both control and Ang II infused animals. Even though both normotensive and hypertensive rats improved their ability to find the hidden platform daily, hypertensive rats had longer path lengths and latency to the platform than normotensive rats, implying that these rats had significantly impaired spatial learning capacity. Moreover, Ang II infused rats pretreated with NaHS significantly improved impairment of spatial learning performance across the 5-day training period. This shows NaHS alleviated the cognitive impairments induced by Ang II induced hypertension.

Given the essential role of synaptic proteins in learning and memory, we examined the expression of key postsynaptic markers in hippocampal synaptosome preparations [31, 32]. Western blotting showed that infusion of Ang II upregulated NMDAR2B, and pretreatment with NaHS markedly reduced this effect, indicating a normalization of the excitatory signaling. We also observed no significant difference in the expression of NMDAR2B between Ang II and NaHS pretreated group (Fig. 2D & F).

Moreover, we also performed western blotting for PSD-95 and synaptophysin, which are proteins of the structural synapses that are important in terms of the synaptic stability and functioning (Fig. 2E). Figure 2G, H indicated that the decreased proteins expression of synaptophysin and PSD-95 in hypertensive rats that were recovered after NaHS pretreatment.

Collectively, these results restoring H_2_S level attenuate AngII- induced dysregulated synaptic protein expression and cognitive dysfunction, showing its neuroprotective potential in hypertension.

### Administration of NaHS attenuates the increase in vessel-associated microglia and microglial activation in angiotensin II infused hypertension

Previous studies have shown association between changes in synaptic proteins, and neuroinflammation which is primarily a cause of cognitive dysfunction. Moreover, neuroinflammatory changes largely arise from endothelial dysfunction, microglial activation, and their complex interaction. Thus, we further sought to understand how microglia respond to Ang II (600 ng/kg/min) for 14 days of induced hypertension. Moreover, to assess the role of H_2_S in hypertension, we also tested whether NaHS-mediated maintenance of H_2_S levels could reverse Ang II-induced hypertension.

**Fig. 3:**
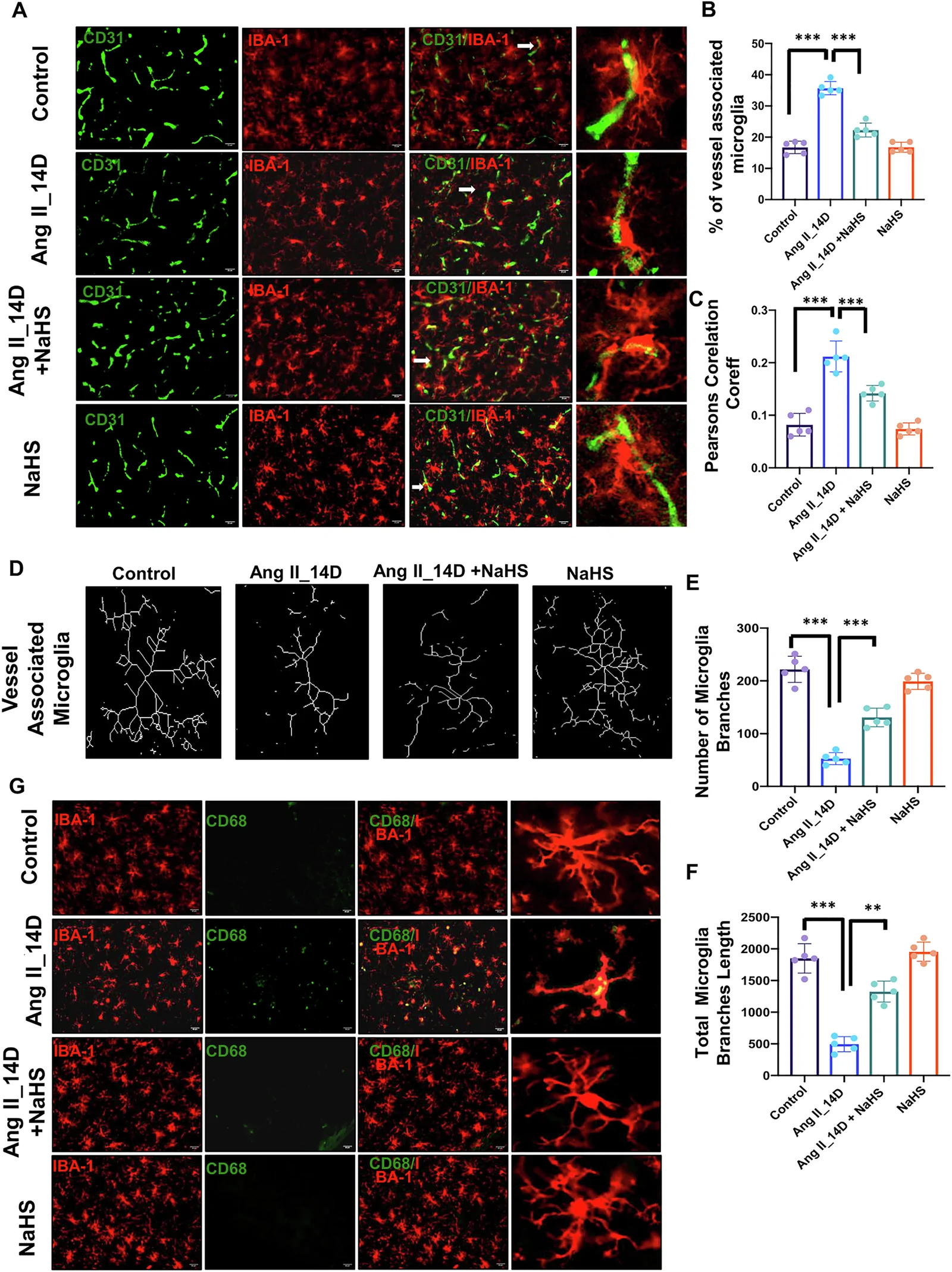
Administration of NaHS attenuates the increase in vessel-associated microglia and microglial activation in Angiotensin II infused hypertension. **A** Representative Co-immunofluorescence of CD31 (endothelial marker, red) and IBA1 (microglia, green) in mentioned groups magnified image shows microglia associated blood vessel region of images Scale bar: 20 μm. **B** Scatter with bar graph represent the percentage of microglia associated with blood vessel (**C**) Scatter with bar graph represents Pearson’s correlation coefficient between IBA1 and CD31 indicating microglia blood vessel overlapping. **D** Microglial morphology quantified by Skeleton analysis using Image J Skeletonize plugin microglia (**E**) Number of microglial branches and (**F**) length of total microglial branch in all groups. **G** Co-immunofluorescence images of IBA1 and CD68 showing CD68 foci in microglia in Ang II induced hypertension group along with other mentioned groups: group 1 is control very provided saline and vehicle, group 2, angiotensin II (600 ng/kg/min) plus vehicle; group 3, angiotensin II plus NaHS (4 mg/kg); group 4 (saline plus NaHS (4 mg/kg) Scale bar: 20 μm Mean ± SEM is shown. *p < 0.05, **p < 0.01, ***p < 0.001.

To evaluate this, we performed co-immunofluorescence labelling of microglia and endothelial cells using IBA-1 (microglial marker) and CD31 (endothelial markers) in the cortical region of the brain (Fig. 3A). We observed a marked increase in the number of microglial associations in the brain blood vessels. The percentage of the microglia associated with a blood vessel within the area observed also increased as calculated by dividing the total number of microglia and vessel-associated microglia (Fig. 3B). We utilized another parameter to determine the colocalization using Pearson’s correlation coefficient, which determines the colocalization of fluorescent pixels between IBA-1 and CD31. The Pearson’s correlation coefficient significantly increased in the Ang II-induced hypertension group compared with control (Fig. 3C). While NaHS treatment prevented Ang II-induced increase in microglia vessel association, as shown by both the percentage of the microglia associated with blood vessels and the Pearson’s correlation coefficient.

Next, we evaluated the morphology of the vessels-associated microglia using skeleton analysis as an indirect measure of microglial activation (Fig. 3D). The quantification of the morphological analysis revealed an activated microglia phenotype with a significantly reduced number of branches and total length of the microglia compared to the control group in vessel-associated microglia in Ang II induced hypertension. NaHS pretreatment under these conditions markedly reversed these effects (Fig. 3E, F). We further strengthened our observation of activated microglia by performing co-immunofluorescence staining of CD68, a lysosomal marker that indicates activated microglia, and IBA1 to stain microglia. We distinctly observed an increase in CD68 dots in the microglia. This analysis shows lower CD68 dots in the microglia in NaHS pretreatment in both Ang II infusion and vehicle groups. This aligns with the morphological changes in activated microglia as observed in the previous experiment (Fig. 3G).

It clearly shows increase in vessel-associated microglia in the Ang II (600 ng/kg/min) for 14 days induces a deleterious microglial phenotype which can exacerbate pathology in already activated endothelium under these conditions and NaHS pretreatment significantly improves Ang II-induced vessel associated microglia morphology and percentage by maintaining H_2_S level.

### NaHS pretreatment exhibits anti-inflammatory effects by modulating cytokine and chemokine profiles in angiotensin II-Induced neuroinflammation

The increased expression of cytokines and chemokines is closely associated with microglial activation, the migration of vessel-associated microglia, and the amplification of neuroinflammatory responses [33, 34]. Consequently, we next investigated how pretreatment of NaHS in attenuating the Ang II Hypertension-induced modulation of cytokines and chemokines. To test this, hippocampal tissue lysates were obtained from control, 14 days Ang II-infused, NaHS-pretreated, and subjected to a cytokine/chemokine protein array assay.

**Fig. 4:**
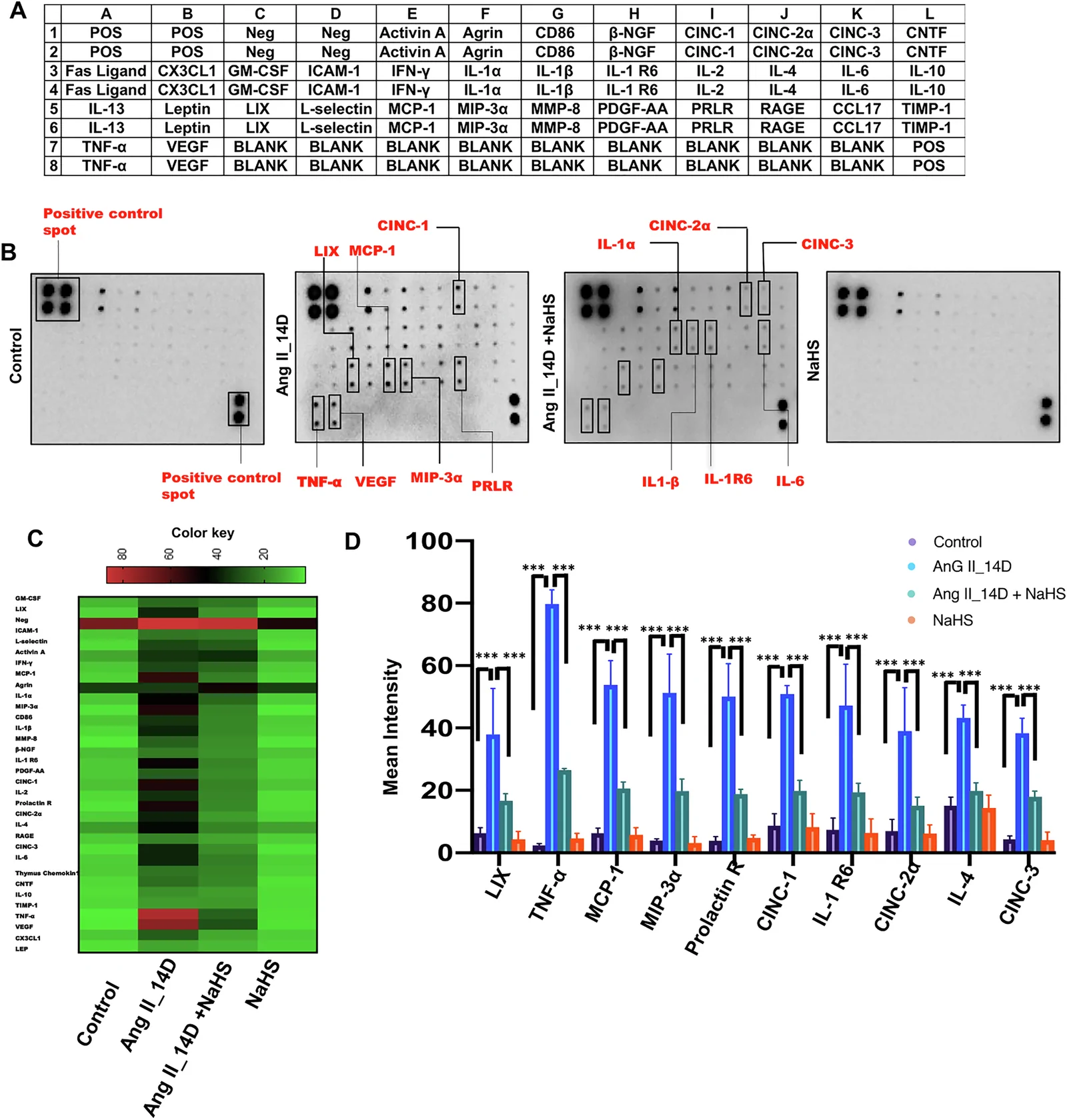
NaHS Pretreatment Exhibits Anti-inflammatory Effects by Modulating Cytokine and chemokine Profiles in Angiotensin II-Induced Neuroinflammation. **A** The layout of protein probed in cytokine and chemokine array along with positive control indicated as POS, each cytokine was detected in duplicate. **B** Representative images showing developed Cytokine and Chemokine arrays for control, Ang II induced hypertension with or without NaHS pre-treatment. Significantly modulated cytokines by NaHS pretreatment are denoted in the box. **C** Heat map illustrating the expression of cytokinesand chemokine in each group. Red and green colours represent relative high and low cytokine expression values, respectively. **D** The quantitative analysis of the cytokine was performed by densitometry using ImageJ. The data represents mean ± SEM. *P < 0.05, **P < 0.01, ***P < 0.001 based on ANOVA; (*n* = 3) biological replicates.

Expressions of 34 cytokines and chemokines were measured by the signals of chemiluminescence then statistically analyzed. The array layout (Fig. 4A) and representative developed cytokine and chemokine array membranes in each experiment group (Fig. 4B) are shown. The produced expression profiles were mapped as a heatmap (Fig. 4C), with color gradients of red and green indicating the highest to lowest protein expression. Multiple cytokines and chemokines were found to be significantly overexpressed in hippocampal lysates of rats with Ang II-induced hypertension, some of these molecules related to neuroinflammation.

Following 14 days of Ang II-induced hypertension, elevated levels of pro-inflammatory cytokines, including TNF-α, VEGF, Neg, IL-1B, Agrin, Activin A, IL-1ɑ were detected in the hippocampus. Additionally, increased expression of another key chemokine such as, CINC-1 (CXCL1), MIP- 3ɑ (CCL20) MCP-1. This further underscore the prevalence of neuroinflammatory condition in hypertension. Notably, these cytokines and chemokines play an important role in neuroinflammation by driving microglial migration and activation.

The pretreatment with NaHS moderated their expression to a significant extent in these conditions, which suggests its anti-inflammatory effects The top ten differentially regulated cytokines in the Ang II group that were significantly modulated by NaHS pretreatment are shown as scatter plots with bar graphs (Fig. 4D). The bar graph shows cytokine expression TNF- ɑ, VEGF, IL-1B, IL 1R6, and chemokines, including CX3CL1, LIX (CXCL), MCP- 1 (CCL2), MIP- 3ɑ (CCL20), CINC-1 (CXCL1), and CINC-2ɑ (CXCL2) in all group. Together, these results suggest a significant increase in expression of hippocampal cytokines/chemokines following a 14-days infusion of Ang II. Notably, pretreatment with NaHS inhibited preferentially microglia-related pro-inflammatory mediators, justifying its anti-inflammatory effect in Ang II-mediated neuroinflammatory responses and attributing H_2_S as a regulator of neuroimmune homeostasis.

### NaHS pretreatment reduces NOX2-Mediated endothelial dysfunction in the hippocampus without modulating AT-1

**Fig. 5:**
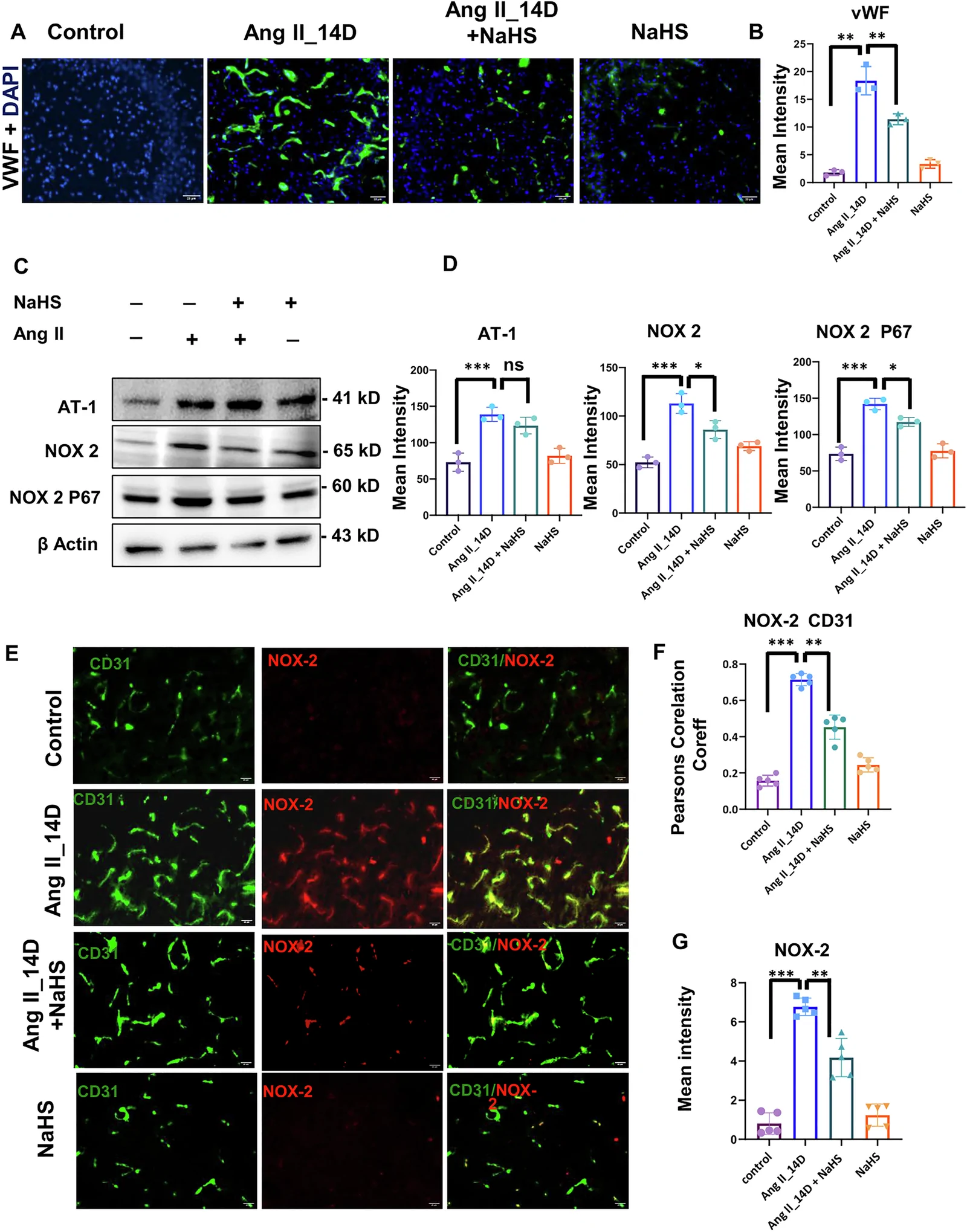
NaHS Pretreatment Reduces NOX2-Mediated Endothelial Dysfunction in the Hippocampus without modulating AT-1. **A** Representative immunofluorescence micrographs showing vWF in hippocampus section Scale bar: 20 μm (**B**) Scatter with Bar graph showing fluorescent signal estimated employing intensity thresholding method in Image J, NIH software. **C** Representative western blots of AT1 (angiotensin II type 1 receptor; AT1R), Nox2, Nox2 p67 in brain tissues of animals. The protein expression signals were normalized to actin (loading control) corresponding scatter with bar graphs showing change in mean intensity (expression) (**D**) AT1, Nox2 and Nox2 p67. (*n* = 3). **E** CO-Immunofluorescence on cortical sections Nox2 (red); cerebral vessels were labeled with CD31(Green) Scale bar: 20 μm. **F** Scatter with bar graph represents Pearson’s correlation coefficient between NOX2 and CD31 indicating microglia blood vessel overlapping (**G**) Corresponding bar graphs showing mean fluorescent intensity of NOX2 (Mean ± SEM) Scale bar: 20 μm. *n* = 3 individual brains and 3 sections/per brain. Statistical analysis was performed using 1-way ANOVA followed by Bonferroni’s post hoc tests. *p < 0.05, **p < 0.01, ***p < 0.001.

Previous studies have shown any dysfunction in the cerebral blood vessels also elicits microglial migration and phenotype changes thus we further evaluated the expression of Von Willebrand Factor (vWF) - a known measure of endothelial dysfunction in all groups. We conducted immunofluorescence staining and found that pretreatment with NaHS reduces the expression of vWF in hippocampus endothelial dysfunction in the hippocampus injury caused by Ang II infusion as shown by micrograph and its quantification in bar graph (Fig. 5A, B).

Moreover, Ang II has been reported to act mainly through the angiotensin type 1 receptor (AT1R), a G-protein-coupled receptor, which is known to promote the formation of the reactive ROS through the activation of the NOX 2 and endothelial dysfunction [35, 36]. Notably, NOX2 is known as a primary effector of Ang II- triggered endothelial dysfunction and neuroinflammation [37, 38].

Next, we sought to answer the question of whether protective effect of NaHS is mediated via regulation of NOX2 expression on the endothelium. In this regard, we performed western blot analysis of the brain (hippocampus) tissue homogenates to examine the protein expression of AT1R, NOX2, and one of its core regulatory components, p67 phox (Fig. 5C, D). Although we did not find significant differences in the level of AT1R expression in experimental groups as compared to controls, we did notice a significant rise in the protein expression of NOX2 and p67phox after a 14-day infusion of Ang II. Notably, a significant decrease in the expression of both NOX2 and p67 phox in the brain was observed after NaHS pretreatment. This observation indicates that the protective effect of H_2_S is not associated with the inhibition of AT1R but more probably reflection of the downstream signaling pathway.

Next, we wanted to know whether NOX2 was expressed in a brain endothelial cell. We performed co-immunofluorescence using NOX2 and CD31, an endothelial marker in the hippocampus region in all groups. We observed significant increase colocalization of NOX2 with CD31 in the Ang II induced hypertension followed by a reversal decrease at NaHS pretreatment group as shown (Fig. 5E). The representative co -immunofluorescence image and Pearson’s correlation bar plot is shown in (Fig. 5E, F). Moreover, increase in the NOX2 protein levels in the brain endothelium was observed in Ang II induced hypertension group as compared to controls. While Ang II-mediated upregulation in fluorescence image of NOX2 in endothelial cells was reduced under pretreatment with NaHS (Fig. 5E & G).

In summary, our data indicate that the protective mechanisms of H_2_S appear to be downstream of or independent of AT1R signaling and are probably mediated by inhibition of NOX2 expression activity. The combination of these results suggests that pretreatment with NaHS also has the potential to prevent endothelial dysfunction by suppressing vWF, endothelial NOX2 expression following Ang II infusion.

## Discussion

**Fig. 6:**
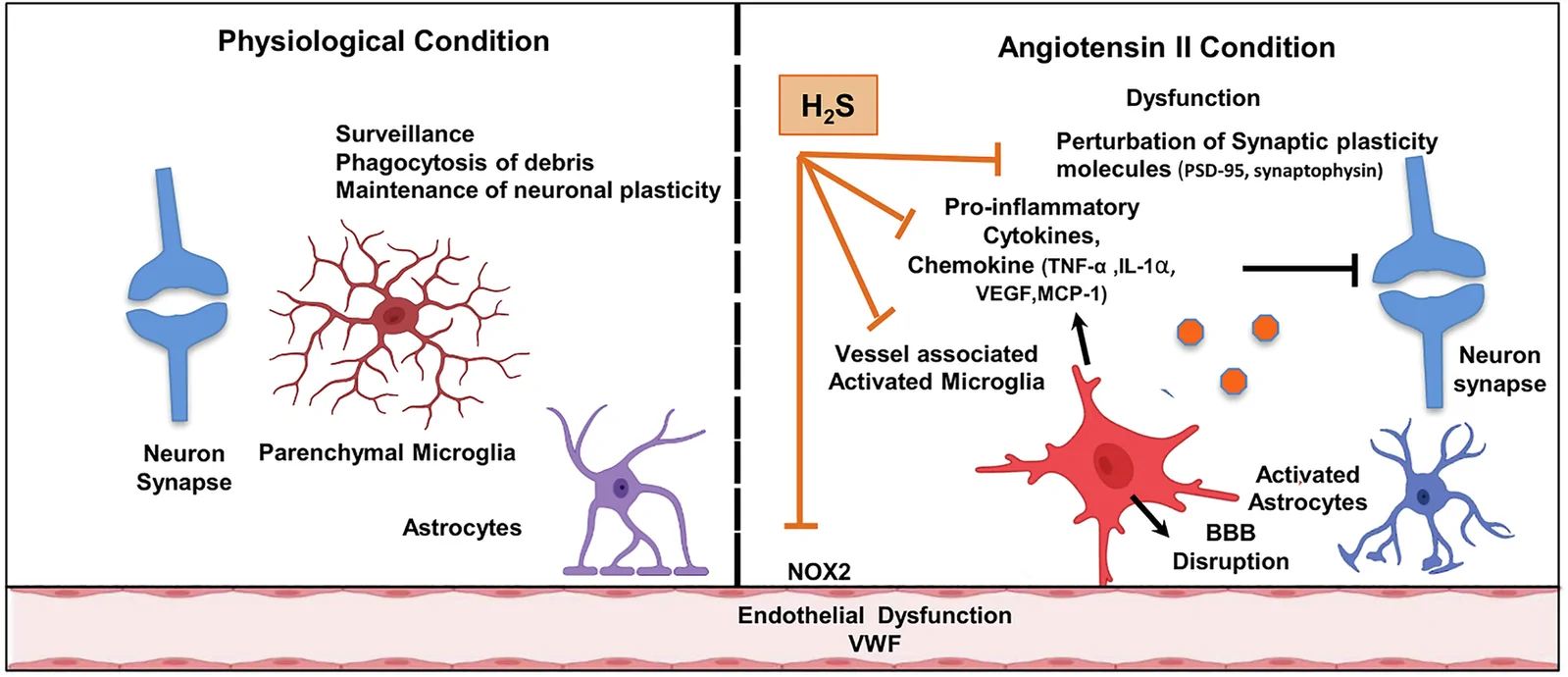
Proposed scheme of events in the present study. A summary figure depicting the neurovascular unit in physiological and Ang II-induced hypertension with NaHS supplementation, which includes astrocytes, pericytes, microglial cells, neurons, and the associated vasculature.In the physiological state, it shows normal microglial morphology, which enables them to survey the physiological status of blood vessels. Ang II-induced hypertension (600 ng/kg/day) causes activation of microglia, an increase in vessel-associated microglia, and endothelial Nox2 activation. Such activated microglia and endothelial cells are associated with upregulation of cytokines and chemokines, which could partially cause the neuroinflammatory milieu and increase endothelial-to-microglial interaction, eventually perturbing neuronal function. Restoration of H_2_S levels via exogenous supplementation NaHS restores endogenous H_2_S levels in the hypertensive brain and curtails microglial endothelial interaction, microglial and endothelial activation, and neuronal dysfunction.

Although large clinical trial like SPRINT-MIND, demonstrate that lowering blood pressure reduces dementia risk, deeper insight into its underlying mechanisms could enable more effective interventions in reducing cerebrovascular complications. Recent studies show that AT₁ receptor blockers reduce brain inflammation, improve vascular function, and provide neuroprotection in models of age-related cognitive decline. This suggests a potential role for AT₁ signaling in cognitive outcomes that may extend beyond blood pressure control. In our study, we observed that Ang II-induced hypertension is associated with significant impairment in spatial learning, accompanied by endothelial dysfunction, microglial activation, increase in vessel-associated microglia, and neuroinflammation. Notably, pre-treatment with NaHS partly restored the normal ramified shape of microglia and vessel-associated microglia. It reduced the release of pro-inflammatory cytokines and chemokines, decreased endothelial NOX2 expression, and partially preserved spatial learning in Ang II-treated rats by modulating synaptic proteins expression. Schematic representation of our observations is summarized in (Fig. 6).

Our study highlighted microglia response to vascular dysfunction in the perpetuation of neuroinflammation, a possible target to reduce the adverse effect of neuroinflammation on brain cognitive ability [37, 39, 40]. Although microglia migration towards the vessel is protective in the early stages, a continuous inflammatory process could have a deleterious effect on blood vessels [17, 41]. Continuous angiotensin II infusion (600 ng/kg/min) for 14 days is most likely exert neuroinflammatory effects on the NVU.The activated microglia could have a double impact on blood vessels and neurons; it could both phagocytize and worsen already hypoperfused blood vessels from microglia-derived proinflammatory cytokines, chemokines and directly influence neuronal activity [42–45]. The previous report of neuroinflammation and changes in microglia morphology and cytokine production in the Ang II slow pressor model also complements our observations [46]. Our study suggests that H_2_S prevents Ang II-induced microglial activation and protects neurons from microglia-mediated damage. Moreover, H_2_S plays a critical role in attenuating Ang II-induced inflammation by reducing microglial migration to the brain vasculature through the modulation of cytokine and chemokine expression under conditions of sustained inflammation. Several cytokine and chemokine identified in our study have previously been shown to regulate microglial activation and migration. For example, TNF- ɑ, VEGF,IL-1B enhance microglial activation and inflammatory signaling, while CINC-1 (CXCL1),MIP- 3ɑ (CCL20), MCP-1(CCL-2) promote directed migration of microglia towards endothelial cells, thereby facilitating their interaction within the NVU during injury and inflammation [47–50]. Our observations are consistent with recent evidence demonstrating that activation of the CCL2/CCR2 pathway promotes microglial migration in chronic hypertension [51].

Furthermore, microglia-mediated cytokine signaling has been shown to influence multiple aspects of synaptic function, including synaptic plasticity, pruning, and transmission [52]. Notably, the cytokines identified in our study could exert a significant impact on the neuronal system. An example is the TNF- α regulates distribution of AMPA receptors [52], and the microglial CX3CR1 with its neuronal CX3CL1 ligand interaction directly contributes to synaptic plasticity [19]. Thus, besides the already established role of vascular impairment, including impairments in cerebral blood flow and neurovascular coupling, the enhanced level of proinflammatory cytokines, and the change in microglial morphology could also be attributed to the alterations in the level of synapse-related proteins and learning capacity observed in these animals [40, 53–57]. Although more electrophysiological studies are required to confirm the observed changes in synaptic function, this study demonstrated that H₂S prevents Ang II–induced microglial activation and protects neurons from microglia-mediated damage [58–62]. In the context of hypertension, these findings suggest that aberrant cytokine-chemokine signaling is not merely a downstream consequence but rather a driving factor that contributes to neurovascular dysfunction, and cognitive impairment. Blood–brain barrier (BBB) permeability was not directly measured in the present study; therefore, we cannot distinguish early Ang II–specific signaling effects from later consequences of BBB dysfunction. Notably, prior studies have demonstrated increased BBB permeability after 14 days of Ang II infusion [24, 63], suggesting a likely role for BBB disruption in the observed neuroinflammatory changes.

In a NaHS pretreatment group in Ang II-induced hypertensive animals, the pro-inflammatory mediators release has been significantly suppressed due to the downregulation of cytokines including TNF-a, VEGF, IL-1a, and IL-1R6. In addition, NaHS supplementation decreased the expression of other chemokines, such as CX3CL1, LIX (CXCL), MCP-1 (CCL2), MIP-3 a (macrophage inflammatory protein 3 a), CINC-1 (CXCL1), and CINC-2 a (CXCL2). Interestingly, in oxLDL-induced inflammation, H₂S inhibits NF-κB p65 by sulfhydrating Cys38, thereby blocking its phosphorylation and binding to the 5′-tri-MCP-1 promoter. This represents a significant effector mechanism of H₂S in the regulation of inflammatory signaling [64]. With conserved cysteine residues of amino terminal of the chemokine subfamilies C, CX3C, CC, and CXC, there is an interest in the scientific community to explore further in terms of understanding direct modulatory effects of H_2_S on these chemokine pathways. Further post-translational studies like protein persulfidation are required to elucidate the mechanistic basis of H_2_S-mediated anti-inflammatory effects [65–67].

Furthermore, recent research has indicated that blocking the activity of NOX2 as a possible treatment protocol would be effective in the treatment of cerebrovascular disease, and in combating the associated cognitive impairments [68–70]. In addition to endothelial dysfunction and microglial activation, activation of NOX2 leads to neuroinflammation through increased release of cytokines and influencing cerebrovascular damage caused by hypertension [57, 71]. NOX2 produces O₂⁻ and H₂O₂ [37], which activate NF-κB in macrophages and promote microglial proliferation. This process increases neuroinflammation and oxidative damage and stimulates NF-κB, HMGB1, inflammasomes, IL-1β, and MMP9 [72–75].

Consistent with these observations we also found that the endothelial NOX2 is more expressed in the hippocampus of Ang II-induced hypertensive animals and NaHS pretreatment inhibited this upregulation. It is plausible that endothelial NOX2 activity represents a critical redox substrate driving neuroinflammatory processes. Most likely continuous interplay between oxidative damage and inflammation contributes to the initiation and progression of hypertension-induced pathology. Several studies have demonstrated an induction of pro-inflammatory cytokines and ROS within NVU by Ang II [76, 77]. Recent evidence has shown that AT1 receptor blocking reduces inflammation, can improve vascular function and helps in the protection of neurons in cognitive decline models [78–80]. Although our data indicate endothelial-associated NOX2 modulation, NOX2 is also expressed in microglia and other neural cell types. Therefore, while endothelial NOX2 may represent a significant contributor in this model, the relative contribution of other cell types requires further investigation [71, 81].

In addition, chronic oxidative damage along with microglial activation as well as blood-brain dysfunction triggers more inflammatory cascades and consequently the risk of cognitive impairment in HTN [82, 83].The possible mechanism of H₂S protection may involve elevation of intracellular NAD⁺ levels and transfer of electrons to mitochondrial Complex II. In addition, H₂S can modify thiol groups of specific cysteine residues in target proteins through sulfhydration, following the increase in intracellular NAD⁺[30, 84–86]. In addition, it enhances the antioxidant defense system through which it regulates the expression of anti-oxidative enzymes that include catalase, superoxide dismutase, glutathione-peroxidase, and glutathione-S-transferase [87]. It is likely that the positive impact of H_2_S in the brain during Ang II-induced hypertension is due to the pleiotropic effects that it acts at multiple pathways and has varied impacts [88]. Thus, H_2_S supplementation offers a promising strategy, to simultaneously inhibit NOX2 activation and attenuate neuroinflammatory signaling. Our findings of brain H_2_S replenishment in Ang II-induced hypertension represents an endogenous mechanism aimed at preventing hypertension-induced neuroinflammatory changes in microglia secondary to vascular dysfunction or damage.

A key challenge of H₂S supplementation is its short half-life [89]. Its effects may persist through sulfane sulfur pools and protein persulfidation [67, 84], but the duration of these effects was not directly measured in this study. While pretreatment of NaHS restored brain H₂S levels and attenuated the rise in blood pressure, it did not fully normalize it, and a blood pressure–matched antihypertensive control group was not included. Therefore, pressure-dependent and Ang II–specific AT₁ receptor–mediated mechanisms could not be clearly separated in this study. More work is required to define the relative contribution of central versus peripheral pathways. Previous work and the current work face a common challenge in clearly distinguishing sustained redox signaling effects from transient sulfide availability [84].

In addition, the chronic Ang II infusion model represents a RAS-dominant and pharmacological form of hypertension [24] that does not fully recapitulate the slow, multifactorial progression of human essential hypertension, thereby limiting translational generalizability [9, 57]. Species, sex, and dose-related differences may further affect reproducibility in Ang II model. Furthermore, the absence of cell-specific mechanistic approaches—such as targeted manipulation of endogenous H₂S-producing enzymes (e.g., CSE) [90, 91] or in vitro validation in isolated neurons and microglia—limits precise understanding of central H₂S signaling pathways. Further studies will ensure clearer mechanistic insight by incorporating blood pressure–matched controls, complementary models such as spontaneously hypertensive rats [92], and cell-selective genetic or viral strategies.

## Conclusion

This study suggests that Ang II–induced hypertension impairs spatial learning and promotes neuroinflammation through microglial activation, enhanced endothelial–microglial interactions, endothelial activation, and a concomitant decrease in H_2_S levels. Pretreatment with NaHS attenuated microglial activation and endothelial dysfunction, reduced hippocampal proinflammatory cytokines and chemokines, preserved spatial learning, and restored synaptic proteins. These effects were mediated by suppression of endothelial NOX2 and subsequent endothelial–microglial interactions.

Collectively, our findings highlight the neuroprotective potential of restoring H₂S levels and demonstrate its multifaceted role in preventing hypertension-induced cognitive impairment and neuroinflammation.

## Data Availability

The datasets generated and analysed during the current study are available from the corresponding author upon reasonable request.
